# Quantification and characterization of the 5′ exonuclease activity of the lysosomal nuclease PLD3 by a novel cell-based assay

**DOI:** 10.1074/jbc.RA120.015867

**Published:** 2020-12-10

**Authors:** Cedric Cappel, Adriana Carolina Gonzalez, Markus Damme

**Affiliations:** Biochemical Institute, Christian-Albrechts-University of Kiel, Kiel, Germany

**Keywords:** lysosomal glycoprotein, nucleoside/nucleotide metabolism, substrate specificity, toll-like receptor (TLR), 5′ exonuclease, fluorescence-quenched oligonucleotide, PLD3, PLD4, AD, Alzheimer’s disease, EFQO, end-labeled fluorescence-quenched oligonucleotide, FAM, 6-carboxyfluorescein, ODN, oligodeoxynucleotide, PAGE, polyacrylamide gel electrophoresis, PTO, phosphorothioate, ssDNA, single-stranded deoxyribonucleic acid

## Abstract

Phospholipase D3 (PLD3) and phospholipase D4 (PLD4), the most recently described lysosomal nucleases, are associated with Alzheimer’s disease, spinocerebellar ataxia, and systemic lupus erythematosus. They exhibit 5′ exonuclease activity on single-stranded DNA, hydrolyzing it at the acidic pH associated with the lysosome. However, their full cellular function is inadequately understood. To examine these enzymes, we developed a robust and automatable cell-based assay based on fluorophore- and fluorescence-quencher-coupled oligonucleotides for the quantitative determination of acidic 5′ exonuclease activity. We validated the assay under knockout and PLD-overexpression conditions and then applied it to characterize PLD3 and PLD4 biochemically. Our experiments revealed PLD3 as the principal acid 5′ exonuclease in HeLa cells, where it showed a markedly higher specific activity compared with PLD4. We further used our newly developed assay to determine the substrate specificity and inhibitory profile of PLD3 and found that proteolytic processing of PLD3 is dispensable for its hydrolytic activity. We followed the expression, proteolytic processing, and intracellular distribution of genetic PLD3 variants previously associated with Alzheimer’s disease and investigated each variant's effect on the 5′ nuclease activity of PLD3, finding that some variants lead to reduced activity, but others not. The development of a PLD3/4-specific biochemical assay will be instrumental in understanding better both nucleases and their incompletely understood roles *in vitro* and *in vivo*.

Lysosomes are central catabolic organelles mediating the hydrolytic degradation of all major classes of cellular metabolites, including proteins, complex lipids, oligosaccharides, and nucleic acids delivered by endocytosis and autophagy. This hydrolytic capacity is maintained by the concerted action of a set of ∼60 mostly soluble enzymes with an acidic pH optimum (1, 2). The great majority of lysosomal enzymes' function is known based on diseases caused by genetic mutations that lead to ∼50 lysosomal storage disorders (1, 2). The function of some lysosomal enzymes, however, is still insufficiently understood.

The degradation of nucleic acids in lysosomes is poorly understood, even though Christian de Duve has already recognized lysosomal nucleases in the earliest description of these degradative organelles (3). DNase II (encoded by *DNASE2A* in humans) is a soluble glycoprotein that acts as an acid endonuclease cleaving double-stranded deoxyribonucleic acid (DNA) with low sequence specificity (4). By analyzing *Dnase2a* knockout mice, its primary physiological function was found to be the degradation of exogenous DNA mainly encountered by phagocytosis of fragmented DNA during cell death (4, 5, 6). Essential functions of DNase II have also been assigned to efficient DNA digestion for the regulation and prevention of aberrant toll-like receptor 9 (TLR9) signaling (7, 8). RNaseT2 is another lysosomal endonuclease that cleaves single-stranded ribonucleic acid (RNA) into mono- or oligonucleotides with generally little sequence specificity but an *in vitro* preference for polyA and polyU, compared with polyG or polyC oligonucleotides (9, 10). In humans, pathogenic mutations in *RNASET2* lead to familial cystic leukoencephalopathy (11). *RnaseT2* mutant zebrafish mirror signs of the human disease and accumulate ribosomal RNA in lysosomes (12).

Other lysosomal nucleic-acid-degrading enzymes are only poorly characterized, and their physiological functions are still largely unknown. Already in 1968, Bernardi *et al*. (13) purified a glycosylated enzyme from bovine spleen, which efficiently cleaved single-stranded DNA (ssDNA) from the 5′ end at an acid pH optimum but double-stranded DNA (dsDNA) only at a much lower efficiency. This enzyme was later assigned as “spleen exonuclease,” but the underlying gene was not cloned or identified (13). Recently, two proteins from the phospholipase D (PLD) family were found to exhibit such ssDNA acid 5′ exonuclease activity: Phospholipase D3 (PLD3) and phospholipase D4 (PLD4). In fact, PLD3 was detected in commercial preparations of spleen exonuclease, implicating that PLD3 alone or both PLD3 and PLD4 represent the spleen exonuclease activity already described by Bernardi and Bernard (14).

Both PLD3 and PLD4 are essential for the efficient hydrolysis of short oligodeoxynucleotides (ODN) in murine immune cells. They prevent the activation of intracellular TLRs, including TLR9, thereby constituting a critical component of the innate immune system (14). Besides its role in the innate immune system, PLD3 previously received considerable attention, as genetic variants in PLD3 were shown to increase the risk of developing Alzheimer’s disease (AD) (15). It should be noted that *PLD3* is highly expressed in the brain and, in particular, in cortical neurons (16, 17). Particularly one coding variant (V232M) was shown to double the risk to develop the disease, and several other variants were exclusively found in AD patients but absent in nondemented control subjects (15). However, this genetic association was challenged later and was only partially or not reproducible at all (18, 19, 20, 21, 22). Whether *PLD3* is indeed an AD risk factor is still controversial. Moreover, how *PLD3* or genetic variants functionally contribute to the AD-linked pathogenic processes has not been determined, and the consequences of the AD-linked genetic variants on the enzyme’s function are still unknown. In addition to genetic variants in AD, a mutation in *PLD3* was recently found to cause for a rare form of autosomal dominant spinocerebellar ataxia (23). As in the case of AD, this genetic association was also challenged because *Pld3* knockout mice do not develop any signs of spinocerebellar ataxia (17).

Except for its function as an ssDNA 5′ exonuclease, little is known about PLD4. *PLD4* is highly expressed in microglia, and it localizes to microglial phagosomes, but it is also found in other tissue macrophages and myeloid cells (14, 24). Mutations in *Pld4* are causative for a hereditary autosomal recessive disease in cattle known as “Bovine hereditary zinc deficiency.” Affected calves suffer from severe skin lesions and show a poor general health status (25). Interestingly, very recently, *PLD4* was genetically linked to systemic lupus erythematosus in human patients in a genome-wide association study, and *Pld4* mutant mice exhibit an autoimmune phenotype, strengthening the role of PLD4 in innate immunity (14, 26).

Both PLD3 and PLD4 are synthesized as N-glycosylated type II transmembrane proteins (27, 28). We have shown previously that PLD3 is transported to lysosomes by an unconventional pathway that comprises sorting into multivesicular bodies and the endosomal sorting complexes required for transport (ESCRT) pathway (27). After arrival in acidic compartments, PLD3 undergoes proteolytic processing yielding the stable soluble glycosylated luminal domain that contains the putative active site and a short membrane-bound N-terminus that is rapidly degraded (27). Whether this proteolytic processing event affects PLD3 activity remains to be determined.

Here we describe a novel robust, reliable, quantitative, and (semi-)automatable cell-based assay for the specific determination of PLD3- and PLD4-mediated acid 5′ exonuclease activity. We used this assay to characterize both enzymes and investigate the effect of proteolytic processing on PLD3 activity. We were now enabled to quantitatively analyze the processing, intracellular transport, and enzymatic activity of different genetic variants of PLD3 previously linked to AD. In summary, our novel assay forms the basis for better understanding the acid 5′ exonucleases PLD3 and PLD4.

## Results

### Acid 5′ exonucleases can be specifically and quantitatively measured by using fluorophore- and fluorescent-quencher-modified oligodeoxynucleotides

**Figure 1:**
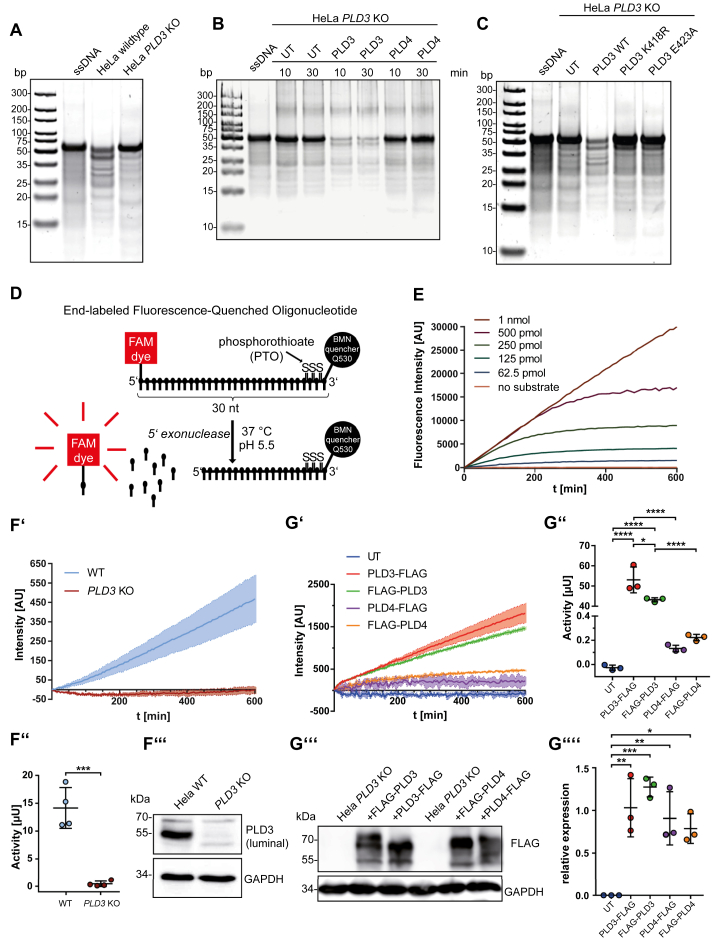
**Testing of cellular-based 5′ exonuclease activity assays and development of a quantitative cell-based assay for acid 5′ exonuclease activity.***A*, ssDNA ODNs separated by PAGE followed by total DNA stain of a 50 nt ssDNA substrate alone, ssDNA treated with cell lysates of wild-type HeLa cells and cell lysates from *PLD3* KO HeLa cells. *B*, ssDNA separated by PAGE followed by total DNA stain of a 50 nt ssDNA substrate alone, ssDNA treated with cell lysates of untransfected (UT) *PLD3* KO HeLa, cell lysates from PLD3-transfected *PLD3* KO HeLa cells, and PLD4-transfected *PLD3* KO HeLa cells. Cell lysates were incubated with the substrate for 10 or 30 min before PAGE. *C*, PAGE of the 50 nt ssDNA substrate alone, ssDNA treated with cell lysates of untransfected (UT) *PLD3* KO HeLa, cell lysates from wild-type PLD3-transfected *PLD3* KO HeLa cells, and *PLD3* KO HeLa cells transfected with the indicated PLD3 mutants. *D*, schematic representation of the EFQO substrate and assay conditions. *E*, released fluorescence over time from cell lysates of wildtype PLD3-transfected *PLD3* KO HeLa cells incubated with the indicated concentration of the EFQO substrate. *F’*, released fluorescence over time from cell lysates of wild-type HeLa cells and *PLD3* KO HeLa cells incubated with 1 μM EFQO substrate. *F’’*, the integrated activity of wild-type HeLa cells and *PLD3* KO HeLa cells. *F’’’*, immunoblot of wildtype HeLa cells and *PLD3* KO HeLa cells used for the enzymatic assay with antibodies against the luminal domain of PLD3 and GAPDH as a loading control. *G’*, released fluorescence over time from cell lysates of 3xFLAG-PLD3 or 3xFLAG-PLD4 transfected *PLD3* KO HeLa cells incubated with 1 μM EFQO substrate. *G’’*, the integrated activity of the released fluorescence shown in *F’*. *G’’’*, immunoblot of the N- and C-terminal FLAG-tagged PLD3-and PLD4-constructs with an antibody against FLAG. GAPDH is depicted as a loading control. *G’’’’*, quantification of the FLAG signals of the immunoblot from independent transfections (N = 3). ∗*p* ≤ 0.05; ∗∗*p* ≤ 0.01; ∗∗∗*p* ≤ 0.001; ∗∗∗∗*p* ≤ 0.0001.

Thus far, the 5′ exonuclease activity of PLD3 and PLD4 has been determined qualitatively or semiquantitatively by incubating the recombinantly expressed purified soluble luminal domains of both PLDs with ssDNA ODNs followed by polyacrylamide gel electrophoresis (PAGE) and total DNA staining (14). To measure nuclease activity in a cell-based assay, *e.g.*, to test for changes in particular genetic variants' activity ectopically expressed upon transfection or endogenous cellular 5′ exonuclease activity, we adapted this assay for cell lysates (Fig. 1). Incubation of total cell lysates from wild-type HeLa cells with a 55 nt ssDNA substrate followed by PAGE revealed efficient substrate cleavage, indicating the assay's suitability to detect endogenous activity in cell lysates (Fig. 1*A*). In contrast, no ssDNA cleavage was observed after incubation of the substrate without cell lysates and negligible cleavage with identical amounts of *PLD3* knockout HeLa cell lysates (27), indicating that PLD3 is the principal acidic 5′ exonuclease in HeLa cells. Transient overexpression of PLD3 was next compared with the overexpression of PLD4, both fused to a FLAG-tag, to compare their specific activity (Fig. 1*B*) directly. While PLD3 efficiently and completely degraded the substrate, only traces of substrate were digested by PLD4 even after 30 min, despite similar expression levels (Fig. S1*A*). We used *PLD3* KO HeLa cells in this and the following overexpression experiments to minimize the contribution of endogenous 5′ exonuclease activity for measuring overexpressed wild-type or mutant PLD3 and PLD4 activities.

Subsequently, we tested if mutations in the catalytic HKD/HKE motifs affect the hydrolytic activity of PLD3 (Fig. 1*C*). PLD3 contains one motif in which the critical aspartic acid (D) is changed to glutamic acid (E) residue (Fig. S1*B*), in contrast to the other members of the PLD-protein family PLD1 and PLD2, which harbor phospholipase D activity but no nuclease activity (15). Mutation of either lysine or glutamic acid in the HKE motif to arginine or alanine in the cDNA, respectively, followed by transfection of *PLD3* KO HeLa cells and the PAGE-based assay revealed a full loss of activity upon mutation of each amino acid, indicating both of them are critical for the nuclease activity and that the HKE motif is critical for catalytic activity (Fig. 1, Fig. S1, *C*–*D*). Ectopic expression of the two mutants in HeLa cells followed by coimmunofluorescence staining with markers for lysosomes (LAMP2) or the endoplasmic reticulum (KDEL) revealed retention of both mutants in the ER (Fig. S1*D*), presumably due to misfolding. This finding is in good agreement with the full loss of function.

The PAGE-based assay did not allow the quantitative estimation of the acidic 5′ exonuclease activity. First, the assay does not measure the activity under substrate saturating conditions, and second, the substrate's disappearance rather than the increase of the product was measured. Prompted by these disadvantages, we developed a quantitative assay for measuring acidic 5′ nuclease activity. A 30 nt long ssDNA substrate with a fluorophore (6-Carboxyfluorescein; FAM) coupled to the terminal 5′ nucleotide and a fluorescent quencher coupled to the terminal 3′ nucleotide (“End-labeled Fluorescence-Quenched Oligonucleotide”; EFQO) was designed (Fig. 1*D*). To prevent 5′ exonuclease-specific cleavage from the 3′-end, the last four 3′ nucleotides were modified by phosphorothioate (PTO), a modification that renders oligonucleotides resistant against the majority of exonucleases (29). The release of the nonquenched 5′-fluorophore-coupled nucleotide, measured by an increase in FAM fluorescence (*e.g.*, in a 96-well-plate fluorescence photometer), reflects a direct and quantitative readout of 5′ exonuclease activity.

We tested the assay's suitability with lysates from HeLa cells transfected with PLD3 (Fig. 1*E*). Equal amounts of lysates were incubated under acidic conditions (pH 5.5) with increasing amounts of the substrate (0–10 μM). Fluorescence, measured over time, showed a substrate-dependent dose response and completed hydrolysis of the substrate at lower substrate concentrations (<10 μM) after incubation for <10 h. Accordingly, fluorescence showed a dose-dependent increase when constant amounts of the substrate (1 μM) were incubated with increasing amounts of cell lysates, indicating an enzyme and substrate dependency following Michaelis–Menten kinetics (Fig. S1*E*). Even with trace amounts of enzyme (100 ng cell lysates in 100 μl reaction volume), the specific activity could be stably measured. In all following assays, the conditions were chosen so that the substrate was in excess by adjusting the amounts of enzyme in the reaction. We applied the EFQO-assay on cell lysates from wild-type HeLa cells and *PLD3* KO HeLa cells (Fig. 1, *F’*–*F’’’*). While readily measurable endogenous 5′-exonuclease activity was observed in the wild-type HeLa cell lysates, activity was below the limit of detection in lysates from *PLD3* KO HeLa cells, validating our findings derived from the semiquantitative PAGE-based assay (Fig. 1*A*). To quantitatively compare the specific activity of PLD3 and PLD4, cell lysates from *PLD3* KO HeLa cells transfected with FLAG-tagged constructs (both enzymes with FLAG at the N- or C-terminus) were measured in the EFQO-based assay (Fig. 1, *G’*–*G’’*). In good agreement with the PAGE-based assay, the specific activity of PLD4 (containing the FLAG-tag at either the N- or C-terminus) was considerably lower compared with PLD3 in the range of ∼300-fold. Protein levels, determined by immunoblot for the FLAG-epitope were, notably, similar between FLAG-tagged PLD3 and FLAG-tagged PLD4 constructs (Fig. 1, *G’’’*–*G’’’’*). Similar data were obtained with untagged constructs, which, however, did not allow direct comparison of the protein levels (not shown). In summary, these data show that PLD3 is the principal 5′ exonuclease in Hela cells and that PLD3 has a significantly higher specific activity compared with PLD4, at least for the tested substrate.

### 5′-PTO-modified phosphodiester bonds protect ssDNA from PLD3/4-catalyzed hydrolysis in cell-based assays

**Figure 2:**
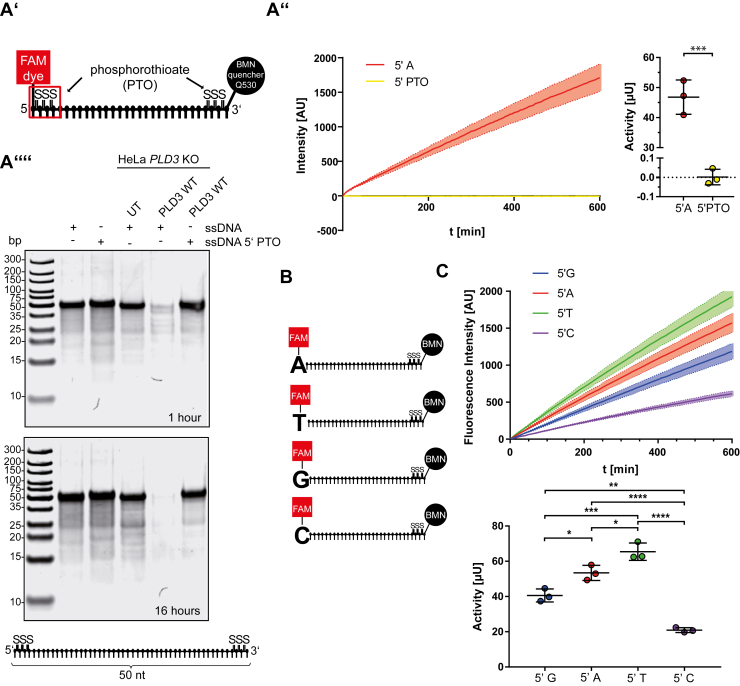
**Determination of the substrate specificity of PLD3 and PLD4.***A’*, schematic representation of the 5′- and 3′-PTO-modified substrate with three PTO-modified internucleotide linkages at the 5′- and 3′-end. *A’’*, released fluorescence over time from cell lysates of untransfected *PLD3* KO HeLa cells and *PLD3* KO HeLa cells transfected with PLD3 with the unmodified substrate (see Fig. 1*D*) and the 5′ PTO-modified substrate. *A’’’*, integrated activity is measured by the EFQO assay of cell lysates from PLD3-transfected cells against the unmodified substrate and the 5′ PTO-modified substrate. *A’’’’*, PAGE assay of untransfected and PLD3-transfected *PLD3* KO HeLa cells incubated with unmodified 50 nt substrate or 5′ PTO-modified 50 nt substrate (depicted below). The substrates were incubated for 1 h (upper panel) or 16 h (lower panel). *B*, schematic representation of the substrates with different terminal FAM-coupled 5′ nucleotides is used to determine the substrate specificity. *C*, integrated activity is measured by the EFQO assay of cell lysates from PLD3-transfected cells against substrates with different 5′-nucleotides. ∗*p* ≤ 0.05; ∗∗*p* ≤ 0.01; ∗∗∗*p* ≤ 0.001; ∗∗∗∗*p* ≤ 0.0001.

PLD3 and PLD4 were qualitatively shown to hydrolyze PTO-modified internucleotide linkages, *i.e.*, the substitution of a sulfur atom for nonbridging oxygen in the phosphate backbone of the oligonucleotide, which renders oligonucleotides resistant to the great majority of nucleases (14). Therefore, we quantitatively compared the specific activity of PLD3 against 5′ PTO-modified or non-PTO-modified oligonucleotides as substrates (Fig. 2*A’*). Cell lysates from *PLD3* KO HeLa cells transfected with PLD3 efficiently released the fluorophore-coupled non-PTO-modified 5′ nucleotide (Fig. 2, *A’’*–*A’’’*). However, they failed to release fluorescence from the PTO-modified 5′ nucleotide above the assay's threshold, indicating cleavage resistance of the PTO-modified substrate. Similar results were obtained with our cell-based PAGE assay: Cell lysates from *PLD3* KO HeLa cells transfected with PLD3 efficiently cleaved the nonmodified substrate after 1 h and quantitatively after 16 h, while the 5′ PTO-modified substrate was not degraded even after prolonged 16 h incubation (Fig. 2*A’’’’*). Next to cell lysates, we used recombinantly expressed PLD3 to test its ability to hydrolyze PTO-modified oligonucleotides in the EFQO assay (Fig. S2, *A’*–*A’’*). Only a marginal turnover of the substrate was detected in comparison to the nonmodified substrate. Increasing the amount of recombinant enzyme by the factor 20 (400 ng instead of 20 ng) showed a tendency toward specific activity against the PTO-modified substrate, which was, however, in the range of 500-fold lower compared with the nonmodified substrate. In summary, our data suggest that PLD3 has only very minor, if at all, activity against PTO-modified substrates under the tested conditions.

### PLD3 has distinct specificities against different 5′ bases

Next, we investigated the substrate specificity of PLD3 regarding the preference of the terminal 5′ nucleotide. Substrates with adenine (A), thymine (T), guanine (G), and cytosine (C) at the fluorophore-coupled 5′-position (Fig. 2*B*) were compared against each other with lysates of HeLa *PLD3* KO cells transfected with *PLD3* (Fig. 2*C*). Under standard conditions, the 5′-terminal nucleotides were released at different rates, with T being released most efficiently, followed by A, G, and C.

### Proteolytic cleavage of PLD3 is dispensable for 5′ exonuclease activity

**Figure 3:**
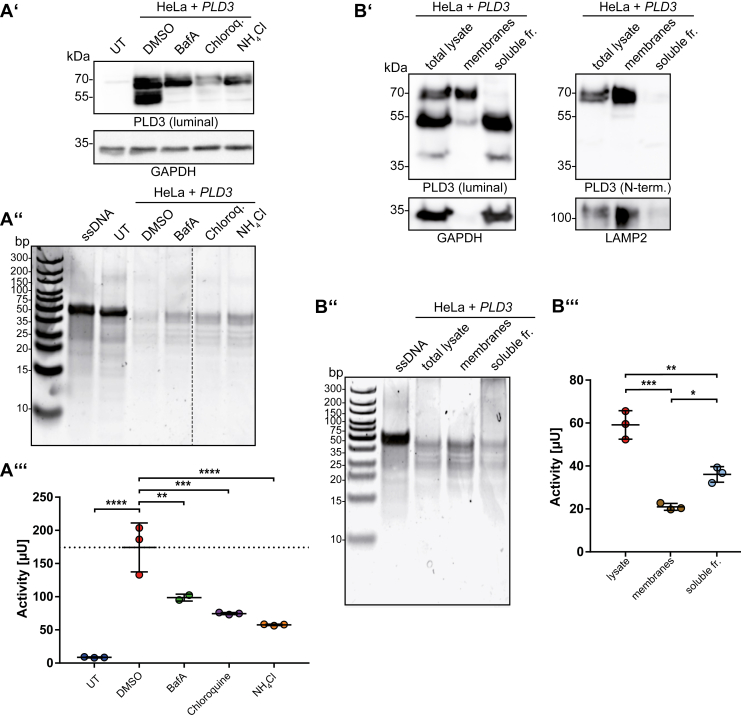
**Proteolytic processing is dispensable for PLD3 activity.***A’*, immunoblot of lysates from untransfected (UT) HeLa cells and HeLa cells transfected with PLD3, treated with lysosomal acidification inhibitors (Bafilomycin A, Chloroquine, NH_4_Cl), with an antibody against the luminal domain of PLD3. GAPDH is depicted as a loading control. *A’’*, ssDNA separated by PAGE followed by total DNA stain of a 50 nt ssDNA substrate alone, ssDNA treated with cell lysates of untransfected *PLD3* KO HeLa cells, and PLD3-transfected HeLa cells treated with inhibitors of lysosomal acidification. *A’’’*, cell lysates' integrated activity treated with the indicated inhibitors of lysosomal acidification measured with the EFQO assay. N = 3. *B’*, immunoblot of total cell lysates, the membrane fraction, and the soluble fraction of HeLa cells transfected with PLD3 with antibodies against the luminal domain of PLD3 or the N terminus of PLD3. Membranes and soluble proteins were separated by ultracentrifugation at 100,000*g*. GAPDH is depicted as a loading control for the soluble fraction; the integral transmembrane protein LAMP2 is depicted as a loading control for the membrane fraction. *B’’*, ssDNA separated by PAGE followed by total DNA stain of a 50 nt ssDNA substrate alone or incubated with aliquots of the total cell lysate from (*B’*), the membrane fraction, or the soluble fraction. Equal amounts of protein were applied for each fraction. *B’’’*, the integrated activity of the total cell lysate, the membrane fraction, and the soluble fraction measured with the EFQO assay. Equal amounts of protein were applied for each fraction. N = 3. ∗*p* ≤ 0.05; ∗∗*p* ≤ 0.01; ∗∗∗*p* ≤ 0.001; ∗∗∗∗*p* ≤ 0.0001.

PLD3 is synthesized as a transmembrane protein that undergoes proteolytic cleavage by acidic pH-dependent protease(s), yielding a soluble luminal enzyme (27). We determined if this proteolytic maturation is a prerequisite for activity or increasing 5′ exonuclease activity, *e.g.*, due to the close proximity to the membrane, which might interfere with ssDNA-substrate accessibility to the catalytic center. To this end, we transfected HeLa cells with *PLD3* and interfered with proteolytic cleavage of PLD3 by treating living cells with inhibitors of lysosomal acidification (Bafilomycin A1, chloroquine, and NH_4_Cl), conditions that effectively inhibit the formation of the soluble form of PLD3 (18) (Fig. 3*A’*). Determination of the specific 5′ exonuclease activity with both the PAGE- (Fig. 3*A’’*) and EFQO (Fig. 3*A’’’*) activity assays *in vitro* revealed reduced 5′ exonuclease activity in lysates of cells treated with all three inhibitors compared with untreated cells. However, this reduced activity is likely a result of a decreased expression or higher turnover of the protein, in other words, less protein, rather than a reduced specific activity (Fig. 3*A’*). In an independent approach, we separated the transmembrane domain-containing full-length protein from the soluble form by ultracentrifugation of total cell lysates from PLD3-transfected HeLa cells (Fig. 3*B’*). Determination of the 5′ exonuclease activity in these fractions with both the PAGE- (Fig. 3*B’’*) and the EFQO-based (Fig. 3*B’’’*) assays again revealed comparable activity of both the full-length protein and the cleaved soluble domain. In conclusion, though not fully quantitative, both experiments confirmed that proteolytic processing is qualitatively dispensable for the 5′ exonuclease activity of PLD3.

### PLD3 does not critically depend on divalent cations and can be efficiently inhibited by vanadate

**Figure 4:**
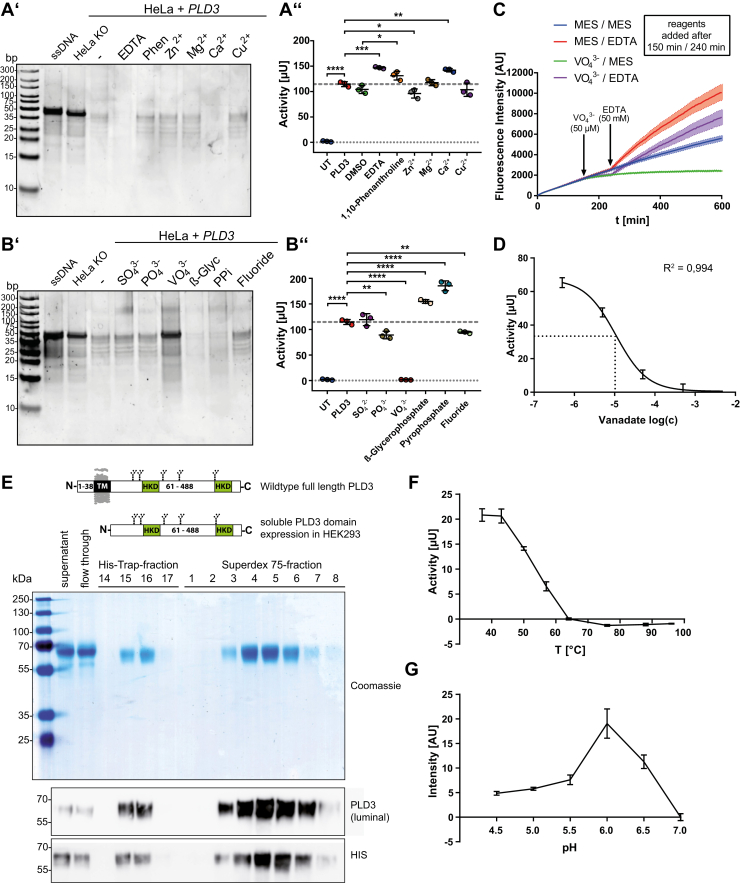
**Inhibitory and activation profile of known nuclease-activity modulators on PLD3 activity.***A’*, PAGE-based assay of *PLD3* KO HeLa cells transfected with PLD3, treated with different chelators (10 mM EDTA; 10 mM 1,10,-Phenanthroline) and divalent cations (1 μM Zn^2+^, 3 mM Ca^2+^, 3 mM Mg^2+^, 1 μM Cu^2+^). *A’’*, EFQO activity assay of lysates treated as analyzed in *A’*. *B’*, PAGE-based assay of *PLD3* KO HeLa cells transfected with PLD3, treated with different anionic phosphatase- and nuclease inhibitors, and phosphate mimetics (10 mM SO_4_^2−^, 10 mM PO_4_^3−^, 500 μM VO_4_^3−^, 10 mM β-Glycerophosphate, 10 mM Pyrophosphate, 2 mM Fluoride). *B’’*, EFQO activity assay of lysates treated as analyzed in *B’*. *C*, EFQO activity assay of *PLD3* KO HeLa cells transfected with PLD3, adding 5 μM Sodium orthovanadate after 2 h and using 5 mM EDTA to revert the inhibiting effect after another hour. *D*, IC50 plot of the inhibitory profile of orthovanadate on PLD3 activity *PLD3* KO HeLa cells transfected with PLD3. *E*, schematic representation of the soluble protein domain expressed in HEK293 cells compared with the full-length PLD3 protein. SDS-PAGE followed by coomassie staining or immunoblots with antibodies against the luminal domain of PLD3, and the His-tag of the different fractions (cell culture supernatant, flow-through, and elution fractions from the Ni^2+^NTA-His-Trap purification and fractions from the Superdex-75 gel-filtration column) from the protein purification are depicted. *F*, temperature-dependent activity plot of the recombinant PLD3 as determined by the EFQO assay. *G*, pH-dependent activity plot of the recombinant PLD3 as determined by the EFQO assay in MES-Buffer containing 20 mM NaCl. ∗*p* ≤ 0.05; ∗∗*p* ≤ 0.01; ∗∗∗*p* ≤ 0.001; ∗∗∗∗*p* ≤ 0.0001.

We next tested the modulating ability of known nuclease- and phosphatase-inhibitors and -activators on the activity of PLD3. The great majority of nucleases critically depend on divalent cations in their catalytic center (30). Therefore, we tested ethylenediaminetetraacetic acid (EDTA) and 1,10-phenanthroline, both chelating unspecifically divalent cations and Zn^2+^ in particular. In independent reactions, divalent cations, including Zn^2+^, Mg^2+^, Ca^2+^, and Cu^2+^, were added to the reaction mixture to test if they could stimulate PLD3 activity. Both chelating agents EDTA and 1,10-phenanthroline slightly but significantly increased instead of inhibiting the 5′ exonuclease activity, as determined by both the PAGE- (Fig. 4*A’*) and the EFQO-assay (Fig. 4*A’’*). Zn^2+^ and Cu^2+^ did not affect PLD3 activity, while Ca^2+^ increased its activity in both the PAGE and EFQO assay. Mg^2+^ slightly increased its activity as detected in the EFQO, but not the PAGE assay. In summary, divalent cations are not essential for the activity, and EDTA and 1,10- phenanthroline can even activate PLD3.

Next, we tested phosphate and different phosphate analogs for their ability to interfere with PLD3 activity (Fig. 4, *B’*–*B’’*). We added sulfate, phosphate, vanadate, β-glycerophosphate, pyrophosphate (PP_i_), and fluoride, which can inhibit a variety of enzymes, to the standard reaction mixture and measured PLD3 activity. Sulfate did not significantly change the activity of PLD3. Phosphate and fluoride slightly reduced its activity. β-glycerophosphate, in contrast, slightly activated PLD3 activity. Vanadate quantitatively inhibited PLD3 activity, as shown by both the PAGE- (Fig. 4*B’*) and the EFQO-assay (Fig. 4*B’’*). The structurally similar orthometalate anions tungstate and molybdate inhibited PLD3 activity but far less efficiently than vanadate (5% and 70% of the inhibition compared with vanadate at 500 μM, respectively) (Fig. S3, *A’*–*A’’*). We next tested whether EDTA can reverse the inhibitory effect of vanadate. To this end, we followed PLD3 activity over time after the addition of a different combination of activators (EDTA) and inhibitors (vanadate) (Fig. 4*C*). As expected, the addition of EDTA stimulated PLD3 activity, while vanadate almost completely inhibited activity. Notably, the addition of EDTA could fully reverse the inhibitory effect of vanadate, indicating that EDTA neutralizes vanadate. Finally, we determined the half-maximal inhibitory concentration (IC50) of PLD3 in cell lysates for vanadate and found that it is a potent inhibitor with an IC50 of 10 μM (Fig. 4*D*).

Next to the cell-based assays, we better characterized the enzymatic activity of PLD3 using the recombinant purified enzyme to avoid background activity and any interfering factors from the cell lysates. For this purpose, we expressed the C-terminally 6xHis-tagged luminal domain of PLD3, starting N-terminally at aa E61 (predicted as the first amino acid after the transmembrane domain) fused to a secretion peptide of IgK (Fig. 4*E*) in HEK 293T cells and selected a stable producer cell line. Soluble PLD3 was purified from the conditioned cell culture medium by Ni^2+^-NTA-resin affinity chromatography, followed by gel filtration (Fig. 4*E*). The heat inactivation of the recombinant protein revealed high stability and a marked decrease in heat stability >50 °C (Fig. 4*F*). The recombinant enzyme's pH-optimum with the EFQO substrate under these conditions was 6.0 (Fig. 4*G*). We also used the recombinant enzyme to test the different divalent cations and EDTA described above in a cleaner experimental setup without the interference of cations or other agents from the cell lysates or the cell culture medium (Fig. S3, *B’*–*B’’*). Mg^2+^ and Ca^2+^ stimulated PLD3 activity, just like that observed with the cell lysates. A combination of both had no additive effect. Compared with the situation observed with cell lysates, EDTA stimulated PLD3 activity, but to a far greater extent (∼5-fold stimulation of recPLD3 *versus* ∼1.4-fold stimulation in cell lysates). Surprisingly, Zn^2+^ and Cu^2+^ efficiently inhibited the recombinant enzyme at high concentrations (Fig. S3, *B’*–*B’’*), but not PLD3 activity in cell lysates (Fig. 4, *A’*–*A’’*), indicating the presence of Zn^2+^/Cu^2+^-neutralizing factors in the cell lysates.

### Genetic variants in *PLD3* affect the proteolytic processing, intracellular sorting, and specific acid 5′ exonuclease activity

**Figure 5:**
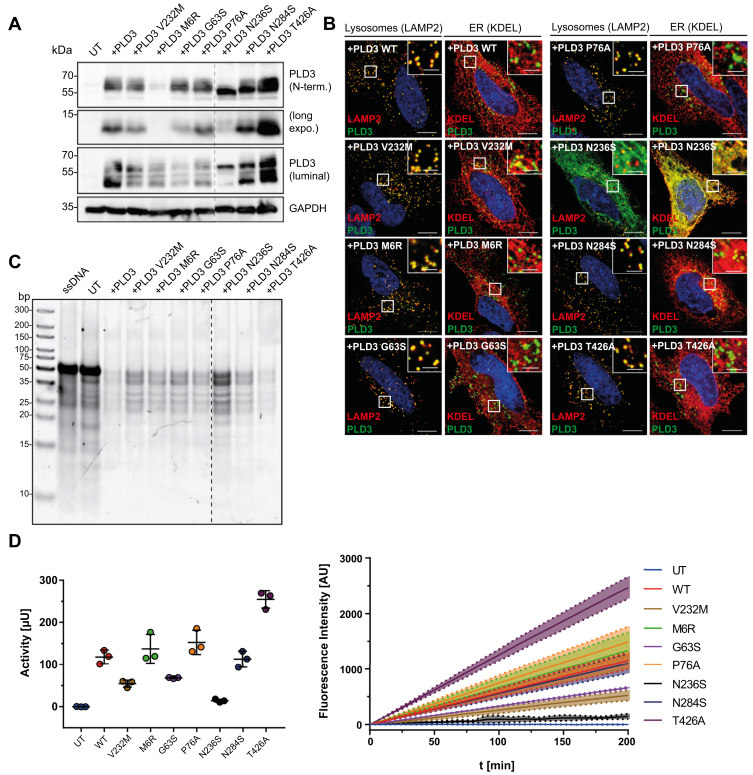
**Effect of genetic variants found in AD patients on expression, subcellular localization, and 5′ exonuclease activity.***A*, immunoblot analysis of cell lysates of HeLa cells transfected with the indicated variants with an antibody against the luminal domain or the N terminus of PLD3. GAPDH is shown as a loading control. The M6R mutation abrogates the N-terminal peptide–antibody binding, which was raised against an epitope in the very N terminus. *B*, coimmunofluorescence staining of each genetic variant transfected in HeLa cells with an antibody against the luminal domain of PLD3 (*green*) and LAMP2 (*red*) as a lysosomal marker or KDEL (*red*) as an ER marker. Nuclei are stained with DAPI (*blue*). *C*, PAGE-based 5′ exonuclease activity assay of lysates from each PLD3 variant expressed in *PLD3* HeLa KO cells. Scale bar large images: 10 μm. Scale bars insets: 2.5 μm. *D*, EFQO-based 5′ exonuclease activity of lysates of each PLD3 variant ectopically expressed in *PLD3* HeLa KO cells.

Genetic variants in *PLD3* have been assigned with a higher risk of developing AD (15, 19, 22), though this genetic association has been challenged later (18, 21, 31). One particular mutation (V232M) was shown to double the risk for developing the disease, and other variants were exclusively found in AD patients but not in healthy nondemented control individuals (15, 19, 22). However, the consequences of these genetic variants on the PLD3 function have not been determined thus far. Therefore, we tested the effect of eight genetic variants (V232M, M6R, G63S, P76A, N236S, N284S, and T426A) in *PLD3* after introducing the mutations in the cDNA by site-directed mutagenesis followed by transfection of HeLa cells and further analysis of PLD3 expression, proteolytic processing, cellular trafficking, and 5′ exonuclease activity (Fig. 5). Immunoblot analysis of the PLD3 variants, with antibodies against the luminal domain and the N terminus, compared with the wild-type protein revealed normal proteolytic processing for all variants except N236S, in which proteolytic maturation was entirely abrogated and both the luminal soluble domain and the small membrane-bound N terminus were not detected (Fig. 5*A*). N284S showed proteolytic cleavage but a slightly reduced apparent molecular weight of the full-length protein, implicating changes in N-glycosylation. T426A was reproducibly expressed more abundantly than the other variants and the wild-type, indicating increased protein stability. Immunofluorescence staining of transfected HeLa cells with PLD3-specific antibodies raised against either the amino-terminus or the luminal domain together with marker proteins for the endoplasmic reticulum (ER; KDEL) or lysosomes (LAMP2) (Fig. 5*B*, Fig. S4) revealed proper sorting of the PLD3 mutants to lysosomes indicated by a high degree of colocalization with LAMP2 for all variants except N236S. This variant showed impaired proteolytic processing (Fig. 5*A*). Lysates of the HeLa cells transfected with plasmids containing each genetic variant were used to determine the specific acid 5′ exonuclease activity by both the PAGE assay (Fig. 5*C*) and the EFQO assay (Fig. 5*D*). The variants M6R, P76A, and N284S did not show any differences in their specific activity compared with the wild-type protein. N236S had only minor residual activity, likely due to misfolding and ER-retention. V232M and G63M showed reduced 5′ exonuclease activity, but both variants were slightly lower expressed compared with the wild-type protein, implicating that the specific activity is either only mildly reduced or similar to the wild-type. T426A showed higher activity compared with the wild-type protein but also significantly higher expression. In summary, genetic variants previously reported being associated with AD lead to a loss of function (N236S), no or only minor loss of function (V232M and G63M), or even gain of function (T426A) in our overexpression- and cell based assays.

## Discussion

The lysosomal catabolic turnover of nucleic acids is still poorly understood, even though nucleases have already been recognized in the earliest description of lysosomes (3). While the essential functions of DNase II in the degradation of exogenous DNA mainly encountered by phagocytosis as well as DNA fragmentation and degradation during cell death and RNaseT2 as the major lysosomal RNA degrading enzyme are well understood (4), the physiological function of other nucleic acid degrading enzymes is just emerging. Especially their role in endo-/lysosomal nucleic acid degradation as an immunomodulatory process for the activation of intracellular toll-like receptors like TLR9 is arising (32). PLD3 and PLD4 are the most recently described lysosomal nucleases that show 5′ exonuclease activity against ssDNA substrates and are critical for balanced endosomal TLR activation (14). In fact such acid 5′ exonuclease activity has been described long time ago and the responsible enzyme was named “spleen exonuclease” (13) and PLD3 is found in commercial preparations of spleen exonuclease (14). However, essential tools to study acidic 5′ exonuclease activity were lacking so far.

We developed a robust, reliable, quantitative, and automatable cell-based assay for acid 5′ exonuclease activity and validated the assay under *PLD3* KO and PLD3/PLD4-overexpression conditions. These experiments revealed that in HeLa cells, virtually all 5′ exonuclease activity arises from PLD3 rather than PLD4. Moreover, our experiments revealed a strikingly higher specific activity of PLD3 compared with PLD4 for the used substrate molecules. Given the predicted high structural similarity between the two enzymes, this finding was surprising. These data implicate either difference for the two enzymes for specific *in vivo* substrate(s), an evolutionary adaption of one of the enzymes for higher substrate turnover, or additional cofactors regulating their function. While *Pld3* and *Pld4* single KO mice show only mild immunological phenotypes, *Pld3/4* double KO mice display a severe phenotype with premature death before 4 weeks of age, implicating high redundancy between the two enzymes and overlapping substrate specificity *in vivo* (14). An explanation for the discrepancy between the two enzymes' specific activities can only be made once natural nucleic acids as endogenous substrates are identified.

The substrate specificity of “spleen exonuclease” regarding the preference of specific nucleotides at the 5′ end was determined previously, however, with conflicting results. In one study, poly(A), poly(I), and poly(U) were hydrolyzed equally well by spleen exonuclease, while poly(C) was resistant to hydrolysis (33). In another study, poly(C) was hydrolyzed to a similar extent compared with poly(U) (34). Presuming PLD3 is responsible for the bulk “spleen exonuclease” activity (14), our data support the finding that internucleotide linkages between a 5′ C-containing nucleotide followed any other nucleotide are relatively poor substrates. Other nucleotides at the 5′ end are hydrolyzed at similar rates. However, it should also be stressed that our EFQO assay offers a greatly improved method to determine this cleavage specificity, as the data derived from previous studies used poly(X) nucleotides, which form secondary structure to a variable degree at acidic pH (35). Poly(C) is a twin-stranded helix at pH 5, possibly explaining its resistance to hydrolysis. However, poly(A) forms similar twin-stranded helices at pH 5 (35). Our assay is independent of any secondary structure as only the hydrolysis of the very 5′ nucleotide phosphodiester bond is measured. Our results reveal that reduced cleavage of cytosine in the 5′ end position is a genuine substrate-nucleotide-specific difference rather than a result of secondary structure.

PLD3 and PLD4 were shown previously to exhibit 5′ activity against PTO-modified substrates (14). In our cell-based-experiments with PLD3 overexpressing cells, we failed to detect significant activity exceeding the assay's background with a 5′ PTO modified substrate. However, we could detect 5′ exonuclease activity against the 5′ PTO-modified substrate with the recombinant purified PLD3-enzyme, like it was used previously (14). However, this activity was only <1% of that of the unmodified substrate, indicating that the value of ∼50-fold lower activity against PTO substrates might have been underestimated with the semiquantitative gel-based assays and that PTO-modification of substrates also confers significant resistance to PLD3 activity.

We also analyzed if proteolytic processing of PLD3 is essential for 5′ exonuclease activity. Our assays, in which we interfered with processing by treating cells with inhibitors of lysosomal acidification or separated both the full-length form and the soluble cleaved luminal form, revealed that full activity is reached even without any proteolytic processing and that even the integral membrane form is active. This finding evokes the question of why proteolytic processing from the integral membrane form to the soluble luminal form occurs. The great majority of lysosomal enzymes are, in fact, soluble, with very few exceptions. A possible explanation for the complex biosynthetic maturation of PLD3 is that it involves an ancient route for its delivery to lysosomes, similar to carboxypeptidase S in the yeast, which involves the membrane-bound cytosolic N terminus (27). Another possibility is that the soluble form of PLD3 has better access to its physiological nucleic acid substrates, which are also soluble in the lysosomal lumen. In living cells, the turnover of these substrates is increased by generating the soluble form over the integral membrane form. The development of an assay suitable for detecting acid 5′ exonuclease activity in living cells could answer this question.

PLD3 and PLD4 bear many similarities compared with the catalytic profile of the *Staphylococcus aureus* nuclease NUC and the eukaryotic mitochondrial PLD6, two members and close homologs of the PLD protein family. The two lysosomal nucleases PLD3/PLD4 and the mitochondrial PLD6 are fully inhibited by orthovanadate and are not affected or even slightly activated by divalent cations Mn^2+^ and Ca^2+^ in the case of PLD6 (36, 37) and Mg^2+^ and Ca^2+^ in the case of PLD3 (Fig. 4). A fascinating observation from our study is that PLD3, very much like NUC and PLD6, not inhibited but even activated by the presence of EDTA and 1,10-phenanthroline, implying that it functions independent of divalent cations. PLD6 contains two Zn^2+^ atoms in a Zinc-binding domain per functional homodimer, critical for ssRNase activity, and speculated to be important for substrate binding (36, 37). EDTA should efficiently chelate this Zn^2+^. We extended our analyses of the PLD3 enzymatic inhibitory profile and included 1,10-phenanthroline in our study, which chelates Zn^2+^ with much higher affinity than other divalent cations. Both PLD3 and PLD4 are still active after the addition of 1,10-phenanthroline, implying minor or no dependence on Zn^2+^. For PLD3 or PLD4, no crystal structure has been solved so far. It will be interesting to compare if PLD3 or PLD4 also contains such critical Zn^2+^ atoms and if they play a significant role in the substrate interaction or any other critical catalytic functions if a structure can be solved. Even though PLD3 and PLD4 show many similarities to PLD6, another striking difference is the strict limitation of the directional cleavage: while PLD6 is a rather unselective 5′ exonuclease processing substrates both in 5′→3′ direction and 3′→5′ direction (36), PLD3 exclusively cleaves nucleotides from the 5′ end (14). On the other hand, NUC cleaves both ssDNA and dsDNA and therefore has a much lower specificity compared with PLD3 and PLD4 (38). These differences are also of significant interest to be compared once a structure of PLD3 and PLD4 can be determined. Another aspect in this regard is the function of the catalytic HKD/HKE motifs: the D or E in the HKD/E-motif of PLD6 is not directly involved in the hydrolytic catalytic process but stabilizing the tertiary structure of the protein by interaction with the peptide backbone of a close α-helix (37). Gottlin *et al*. (39) describe the same effect also in NUC. Our data showing an ER retention of proteins harboring mutations in the second HKE motif support the similarity of PLD3 to PLD6 and NUC.

PLD3 and PLD4 are critical for regulating lysosomal ssDNA, which activates intracellular TLRs and particularly TLR9 (32). The involvement of both enzymes in autoimmune disorders is therefore conceivable. In this line, genetic variants in *PLD4* have been recently linked to systemic lupus erythematosus in human patients by GWAS (26). Modulating PLD3 and PLD4 activity might be, therefore, an attractive target in autoimmune-targeted therapies. Our assay, which could be easily automated, could be easily used for medium-throughput small drug screening for specific inhibitors and activators for acid 5′ exonuclease activity and even more specifically for PLD3 or PLD4.

We used our assay to investigate the effect of genetic variations in *PLD3*, possibly associated with AD (15). Moreover, genetic variants in *PLD3* have been linked with cerebrospinal fluid total-tau and phosphorylated-tau levels (19) and cognitive function in AD patients (40) and additionally associated with other neurological traits like longevity (41). However, this genetic link between variants in *PLD3* and AD has been discussed controversially, and particularly a direct effect of PLD3 on amyloid precursor protein is questionable (16, 19, 20, 30, 42). Our assay offers the possibility to functionally test coding variants on 5′ exonuclease activity and possibly to test in functional assays how PLD3 might affect nuclease-dependent AD-relevant pathologic pathways. Interestingly, the major coding variant V232M showed reduced activity in our experiments. A mutation in PLD3 has recently been linked to an autosomal dominant form of spinocerebellar ataxia (23), even though it remains questionable if the mutation in *PLD3* is causative for the disease (17). The assay described here might be used as a suitable test for diagnosing this subtype of spinocerebellar ataxia.

## Experimental procedures

### Plasmids, antibodies, cell lines, and chemicals

The cDNA construct for human PLD3 (hPLD3) was previously described (27). The following constructs were generated by site-directed mutagenesis (see below): PLD3 K418R, PLD3 E423A, PLD3 V232M, PLD3 M6R, PLD3 G63S, PLD3 P76A, PLD3 N236S, PLD3 N284S, and PLD3 T426A. To generate FLAG-tagged constructs, we inserted a 3xFLAG sequence (DYKDHDGDYKDHDIDYKDDDDK) into the pcDNA4-TO vector (Invitrogen) using the restriction enzymes HindIII/NotI for the N-terminal tag and NotI/ApaI (all from Thermo Fisher Scientific) for the C-terminal 3xFLAG-tag. We cloned hPLD3 or hPLD4 cDNA into the resulting vectors using NotI plus XbaI to get N-terminally tagged constructs and HindIII plus NotI (for hPLD3) or XhoI (for hPLD4) for C-terminally tagged ones. All constructs were sequence-validated by Sanger sequencing (Eurofins Genomics). Evaluation of transient gene expression was performed by transfection of HeLa cells with polyethylenimine (PEI, Polysciences). Cells were kept at 37 ºC in a humidified atmosphere at 5% (v/v) CO_2_ and processed 24 h posttransfection. For a stable expression system in HEK 293T cells, the hPLD3-cDNA sequence was cloned beginning at position amino acid position 61 and adding an N-terminal IgK-signal peptide and a C-terminal His6-Tag into the pcDNA3.1/TnhEF-SB9-cl1-real vector (gifted by Dr Manfred Gossen). HeLa and HEK 293T cells were purchased from ATCC. The PLD3 N-terminal antibody was generated by immunization of rabbits with the murine PLD3 N-terminal peptide (27). The rabbit polyclonal PLD3 antibody against the luminal domain was purchased from Sigma-Aldrich. GAPDH antibody was purchased from Santa Cruz. The CRISPR *PLD3* knockout cell line was described previously (27). Standard chemicals, if not stated otherwise, were purchased from Sigma-Aldrich.

### Site-directed mutagenesis

For PCR-mediated mutagenesis, primers were designed carrying the desired mutation, including additional 15 to 20 nucleotides up- and downstream the mutation site. PCR was performed using Phusion high-fidelity polymerase in compatible HF-buffer, followed by digestion with 10 U DpnI (both from Thermo Fisher Scientific) for 1 h at 37 °C. Chemo-competent XL-1-blue *E. coli* have been transfected with the obtained PCR products and selected on lysogeny broth (LB) agar plates, treated with ampicillin. For plasmid preparation, mini- and midi cultures were raised in LB-Ampicillin-medium overnight at 37 °C, followed by preparation using the GeneJET Plasmid Miniprep Kit (Thermo Fisher Scientific) for mini- and the PureYield Plasmid Midiprep System (Promega) for midi preparation. For all mutants, pcDNA3.1/Hygro(+) was used as a template vector. Confirmation of the mutation was conducted by Sanger Sequencing using the GATC LightRun service (Eurofins genomics).

### Expression and purification of recombinant human PLD3

Wild-type HEK 293T cells were cotransfected with both the vector containing the desired hPLD3-sequence and the pCMV(CAT)T7-SB100(AL) vector (Dr M. Gossen) containing a Sleeping beauty (SB100) transposase gene. Two days after transfection, the cells were further cultivated in selection medium containing 7 μg/ml Puromycin (InvivoGen). Monoclonal cell lines were further generated by serial dilution in 96-well-plates. Clonal single-cell colonies were tested for expression efficiency using SDS-PAGE and western blot.

The obtained cell lines were grown to ∼90% confluency in T175 cell culture flasks (Sarstedt) under selection conditions for purification. Production medium (100 ml) containing only 2.5% fetal calf serum was added for 7 days and harvested by spinning down cell debris twice for 5 min at 500 g, followed by sterile filtration. The cell culture supernatant was concentrated to a final volume of 50 ml and purified on the AEKTA protein purification system using the HisTrap HP Ni-charged IMAC column (GE Healthcare) and subsequent size-exclusion chromatography using a Superdex 75 column (GE Healthcare) with PBS as the final solvent. The obtained fractions were pooled and concentrated by centrifugation in a Vivaspin 20 ultrafiltration unit (cutoff 10 kDa, Sartorius) for 20 min at 3300*g* and 4 °C. Purity was confirmed by SDS-PAGE and Coomassie stain.

### Immunocytochemistry and microscopy

Semiconfluent cultures of HeLa cells grown on coverslips were fixed with 99% cold methanol or 4% (w/v) paraformaldehyde solution for 20 min at room temperature. After permeabilization and blocking, coverslips were incubated overnight with the indicated primary antibodies. After incubation with fluorophore-conjugated secondary antibodies (AlexaFluor; Thermo Fisher Scientific) and washing, coverslips were embedded in the mounting solution. Images were analyzed with an Olympus FV1000D Laser Scanning Confocal Microscope (Model: FV10-292-115) with a 100x lens (UPLSAPO 100x NA: 1.40). Image acquisition was performed with the FV10-ASW 4.2 Viewer software (Olympus GmbH, Germany).

### Protein extraction and western blot analysis

Protein lysates of cultivated cells for western blotting procedures were performed according to standard protocols described previously (27).

### Polyacrylamide-gels for DNA separation

A qualitative assay for PLD3 nuclease activity was analyzed by denaturing polyacrylamide gel electrophoresis, as previously described by Gavin *et al*., 2018. Briefly, 50 μg of protein lysate was incubated with 5 μM ssDNA (5′-ACCATGACGTTCCTGATGCTAAGTATGCACTTCATCGTCAAGCA ATGCTATG∗C∗A∗T-3′, Biomers) modified with internucleotide phosphorothioate (∗) in MES buffer (50 mM MES pH 4.5, 100 mM NaCl) for the indicated time at 37 ºC. The enzymatic reaction was deactivated by incubation at 96 ºC for 5 min, and samples were mixed with 6X DNA loading dye (Thermo Fisher Scientific). DNA was separated in a vertical chamber using a 20% denaturing polyacrylamide gel in 1X Tris-Borate-EDTA (TBE) buffer (1 M Tris, 1 M Boric acid, 0.02 M EDTA) applying a current of 60 V for 6 h. The GeneRuler Ultra Low Range DNA Ladder (Thermo Fisher Scientific) was used to monitor the progress of the electrophoresis. DNA gel was incubated at room temperature for 5 min in 1X SYBR Gold Nucleic Acid Gel Stain (Invitrogen) prepared in TBE buffer. The acquisition was performed with the Odyssey FC Imaging system at 600 nm and visualized with the Image Studio Software (LI-COR).

### End-labeled fluorescence-quenched oligonucleotide (EFQO) assays

EFQO assays were conducted in a lumox multiwell 96 plate (Sarstedt). If not indicated, otherwise, the standard reaction conditions were at pH 5.5 in MES-buffer containing 50 mM 2-(N-morpholino)ethanesulfonic acid (MES) and 20 mM NaCl. 1 pmol/μl FAM-ssDNA-substrate (Biomers) (Sequence: ACCATGACGTTCCTGATGCTAAGTATG∗C∗A∗C∗ with ∗ indicating a phosphorothioate bond) and 5 ng/μl protein from crude cell lysate (50 ng/μl when using untransfected wildtype cells) were added to a final volume of 100 μl. After a preincubation period of 30 min, fluorescence emission at 528 nm (following excitation at 485 nm) was measured in a microwell plate reader (Synergy HT from BioTek) from below the wells for 12h every 5 min while incubating at 37 °C. For evaluation, a substrate control without lysate and a lysate control without substrate were measured together with the samples. For calculating the resulting activity, the following formula was used:

$$I_{C}\left(t\right)=\;I_{M}\left(t\right)-I_{M}0-\left(\frac{dI_{S}(t)}{dt}\;+\;\frac{dI_{E}(t)}{dt}\right)\times t$$

With: *I*_*C*_*(t)* = calculated sample intensity

*I*_*M*_*(t)* = measured sample intensity

*I*_*M*_*0* = measured sample intensity at t = 0 (after 30 min preincubation)

*I*_*S*_*(t)* = measured intensity of the substrate control

*I*_*E*_*(t)* = measured intensity of the enzyme control

*t* = time

For calibration, different concentrations of substrate were incubated with an excess of recombinant hPLD3 as for an EFQO assay, taking the I_max_ of the resulting graphs as intensity values of a fully digested substrate and calculating a calibration line whose slope corresponds to the proportionality factor *k* to calculate the enzyme activity using the formula:

$$A=\frac{dI_{c}\left(t\right)}{dt}\times \frac{1}{k}$$

With: *A* = activity

*I*_*c*_*(t)* = calculated sample intensity

*t* = time

*k* = fluorescence coefficient (in mol^−1^)

### Quantification and statistical analyses

If not stated otherwise, a one-way-ANOVA using Dunnett’s test to correct for multiple comparisons was calculated using GraphPad Prism Software Version 7.04. Significant values were considered at *p* < 0.05. Values are expressed as mean ± standard error of the mean (SEM) and significance is designated as ∗*p* < 0.05; ∗∗*p* < 0.005; ∗∗∗*p* < 0.0001.

## Data availability

All data, associated protocols, methods, and sources of materials are available in the main text or in the supporting information.

## Conflict of interest

The authors declare that they have no conflicts of interest with the contents of this article.
