# Supplementary material for: Quantification and characterization of the 5′ exonuclease activity of the lysosomal nuclease PLD3 by a novel cell-based assay
Source: J Biol Chem. 2020 Dec 10;296:100152. doi: 10.1074/jbc.RA120.015867 (PMC7857491; doi:10.1074/jbc.RA120.015867)
Supplement: Figures S1–S4 [file mmc1.pdf]

Supporting Information

Quantification and characterization of the 5'exonuclease activity of the lysosomal nuclease PLD3 by a novel cell-based assay

Cedric Cappel[1,†], Adriana Carolina Gonzalez[1,†], Markus Damme[1,#]

† These authors contributed equally to this work

[1] Biochemical Institute, Christian-Albrechts-University of Kiel, Kiel 24118, Germany

SUPPORTING FIGURES

Supporting Information Figure 1

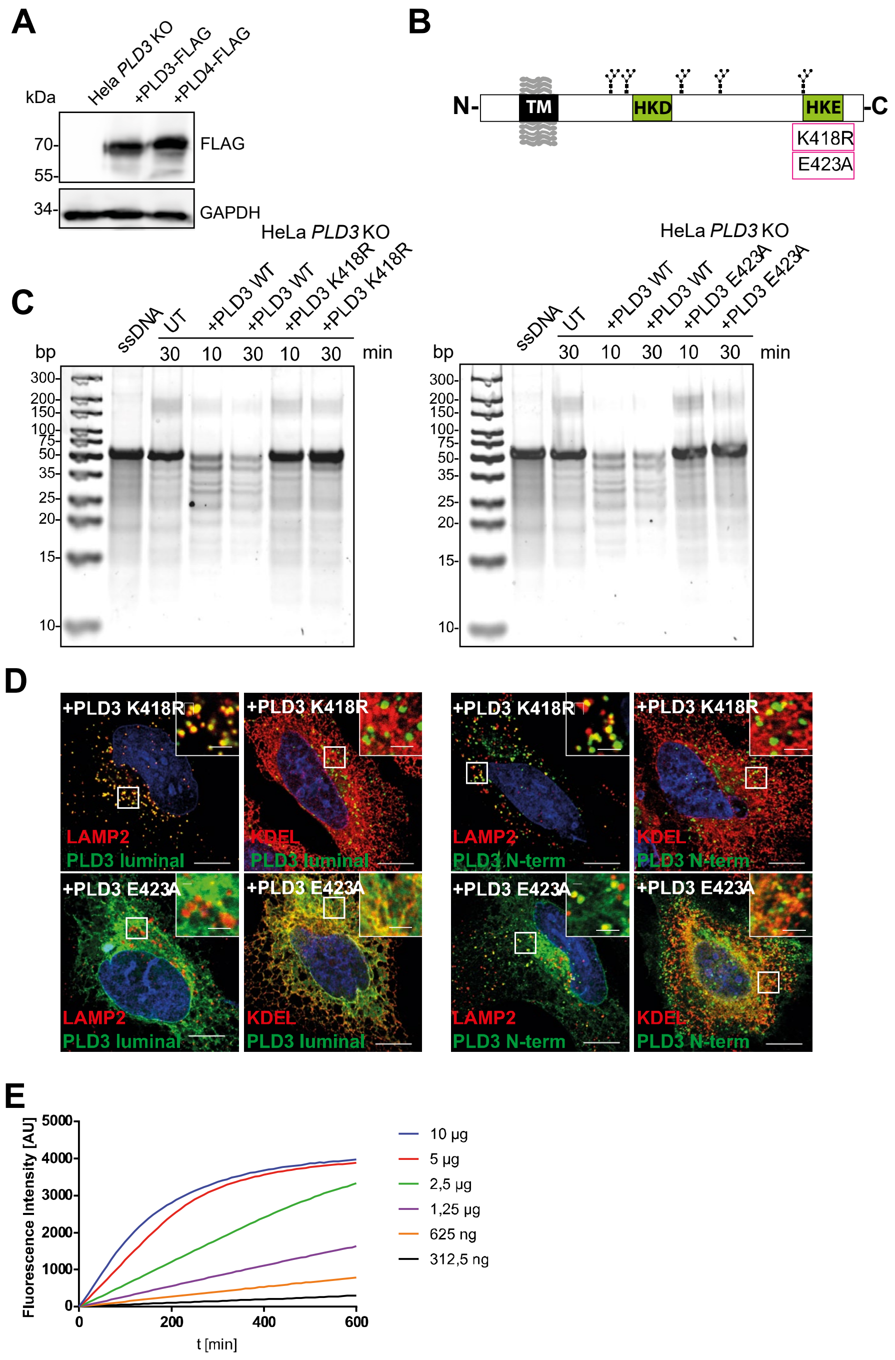

Supporting Information Figure 1. (A) Immunoblot of lysates used for the gel-based activity assay (**Figure 1B**) of C-terminally FLAG-tagged PLD3- and PLD4-constructs with an antibody against FLAG. GAPDH is depicted as a loading control. **(B)** Schematic representation of the domain structure of PLD3. The HKD and HKE motifs are depicted in green. The position of the K418R and E423A-mutations in the HKE motif is indicated. **(C)** PAGE of the 50nt ssDNA substrate alone, ssDNA treated with cell lysates of untransfected (UT) PLD3 KO HeLa, cell lysates from wildtype PLD3-transfected PLD3 KO HeLa cells, and PLD3 KO HeLa cells transfected with the indicated PLD3 mutants. The substrate and cell lysates were incubated for 10 and 30 minutes, respectively. **(D)** Co-immunofluorescence staining of each mutant cDNA (K418R and E423A) transfected in HeLa cells with an antibody against the luminal domain of PLD3 (green) and LAMP2 (red) as a lysosomal marker or KDEL (red) as an ER marker. Nuclei are stained with DAPI (blue). Scale bar large images: 10 µm. Scale bars insets: 2.5 µm. **(E)** Released fluorescence over time with the indicated amounts of cell lysates of wildtype PLD3-transfected *PLD3* KO HeLa cells incubated with the EFQO substrate.

Supporting Information Figure 2

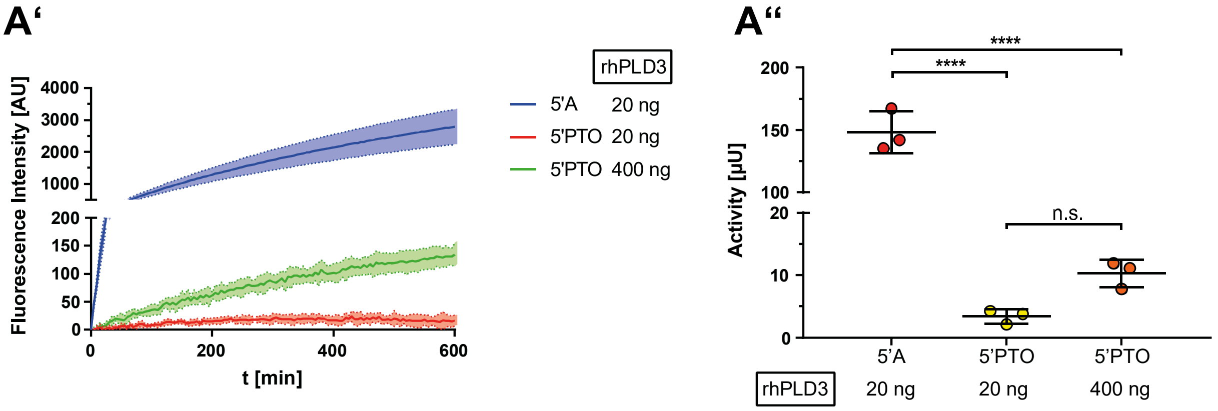

Supporting Information Figure 2. PTO-modified oligonucleotides are poor substrates for PLD3. (A') Released fluorescence over time with fixed amounts of cell lysates of PLD3-transfected *PLD3* KO HeLa cells incubated with the indicated amounts of the non-PTO-modified substrate (5'A) or indicated concentrations of PTO-modified EFQO substrate (5'-PTO). **(A'')** Bar-graph representation of the turnover of the non-PTO-modified substrate (5'A) or indicated concentrations of PTO-modified EFQO substrate (5'-PTO).

Supporting Information Figure 3.

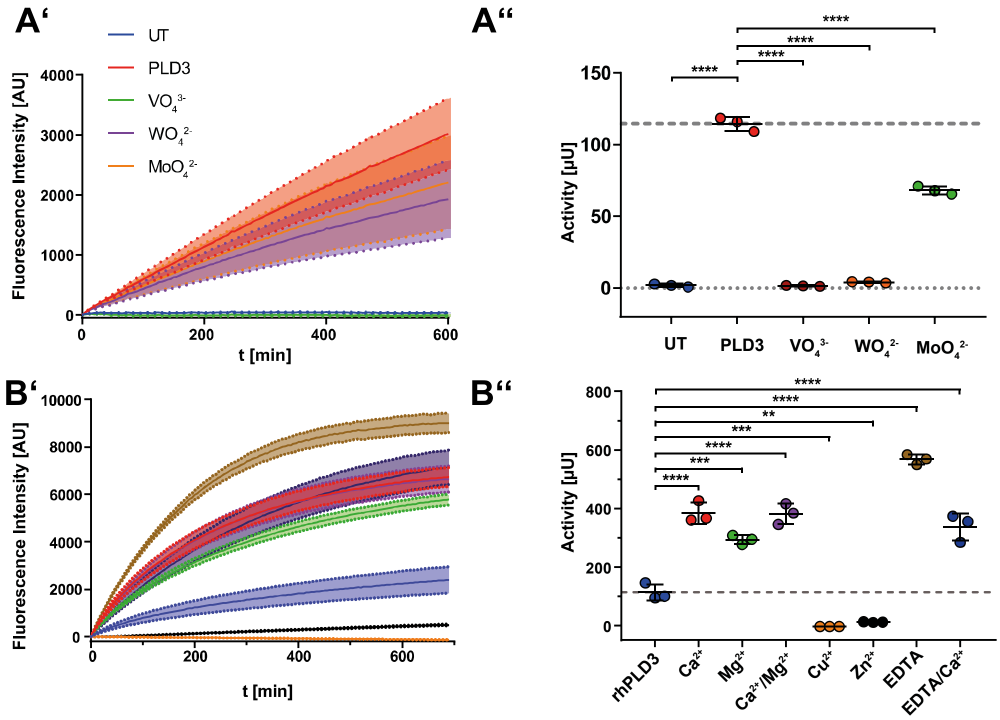

Supporting Information Figure 3. Orthometalate anions, Cu^{2+} and Zn^{2+} inhibit PLD3. (A', A'') EFQO-based assay of PLD3 KO HeLa cells transfected with PLD3, treated with 500 µM of the polyoxyanions vanadate (VO_4^{3-}), tungstate (WO_4^{2-}) or molybdate (MoO_4^{2-}) **(B', B'')**. EFQO-based assay of recombinant PLD3 incubated with Ca^{2+} (3 mM), Mg^{2+} (3 mM), Ca^{2+} + Mg^{2+} (1,5 mM each), Cu^{2+} (1 mM), Zn^{2+} (1 mM) and EDTA (10 mM).

Supporting Information Figure 4

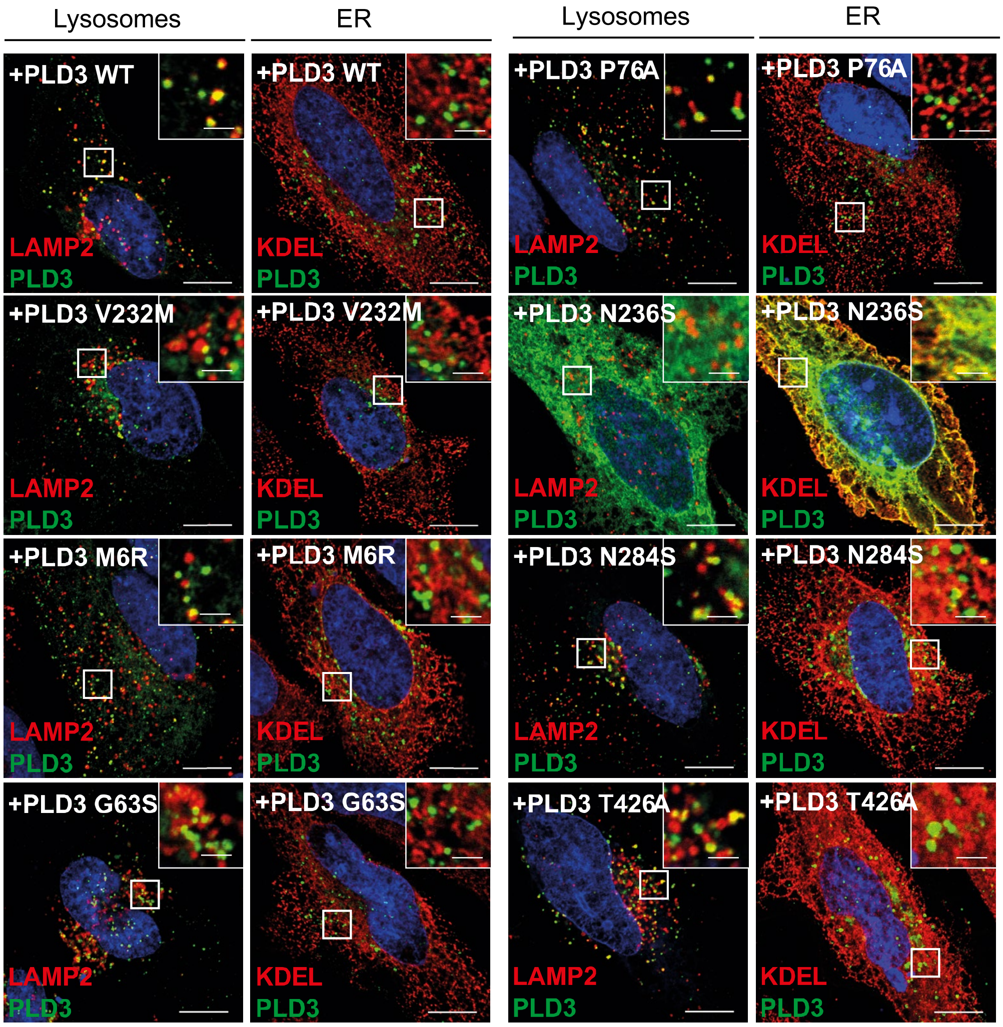

Supporting Information Figure 4. Effect of genetic variants found in AD patients on subcellular localization determined with an antibody against the cytosolic N-terminus. Coimmunofluorescence staining of each genetic variant transfected in HeLa cells with an antibody against the cytosolic N-terminus of PLD3 (green), LAMP2 (red) as a lysosomal marker or KDEL (red) as an ER marker. Nuclei are stained with DAPI (blue). Scale bar large images: 10 µm. Scale bars insets: 2.5 µm.
